# A cyclic nucleotide sensitive promoter reporter system suitable for bacteria and plant cells

**DOI:** 10.1186/1472-6750-13-97

**Published:** 2013-11-09

**Authors:** Janet I Wheeler, Lubna Freihat, Helen R Irving

**Affiliations:** 1 Monash Institute of Pharmaceutical Sciences, Monash University (Parkville campus), 381 Royal Parade, Parkville, VIC 3052, Australia

**Keywords:** Cyclic GMP, Cyclic AMP, Luciferase reporter, OPTX promoter

## Abstract

**Background:**

Cyclic AMP (cAMP) and cyclic GMP (cGMP) have roles in relaying external signals and modifying gene expression within cells in all phyla. Currently there are no reporter systems suitable for bacteria and plant cells that measure alterations in downstream gene expression following changes in intracellular levels of cyclic nucleotides. As the plant protein OLIGOPEPTIDE TRANSPORTER X (OPTX) is upregulated by cGMP, we fused the OPTX promoter to a luciferase reporter gene (OPTX:LUC) to develop a plant cell reporter of cGMP-induced gene expression. We prepared a second construct augmented with three mammalian cGMP response elements (OPTXcGMPRE:LUC) and a third construct containing five gibberellic acid response elements (OPTXGARE:LUC). All three constructs were tested in bacteria and isolated plant protoplasts.

**Results:**

Membrane permeable cGMP enhanced luciferase activity of OPTX:LUC and OPTXGARE:LUC in protoplasts. Treatment with the plant hormone gibberellic acid which acts via cGMP also generated downstream luciferase activity. However, membrane permeable cAMP induced similar responses to cGMP in protoplasts. Significantly increased luciferase activity occurred in bacteria transformed with either OPTXcGMPRE:LUC or OPTXGARE:LUC in response to membrane permeable cAMP and cGMP. Bacteria co-transformed with OPTXcGMPRE:LUC or OPTXGARE:LUC and the soluble cytoplasmic domain of phytosulfokine receptor1 (PSKR1; a novel guanylate cyclase) had enhanced luciferase activity following induction of PSKR1 expression.

**Conclusions:**

We have developed promoter reporter systems based on the plant OPTX promoter that can be employed in bacteria and isolated plant cells. We have shown that it can be used in bacteria to screen recombinant proteins for guanylate cyclase activity as increases in intracellular cGMP levels result in altered gene transcription and luciferase activity.

## Background

**Figure 1:**
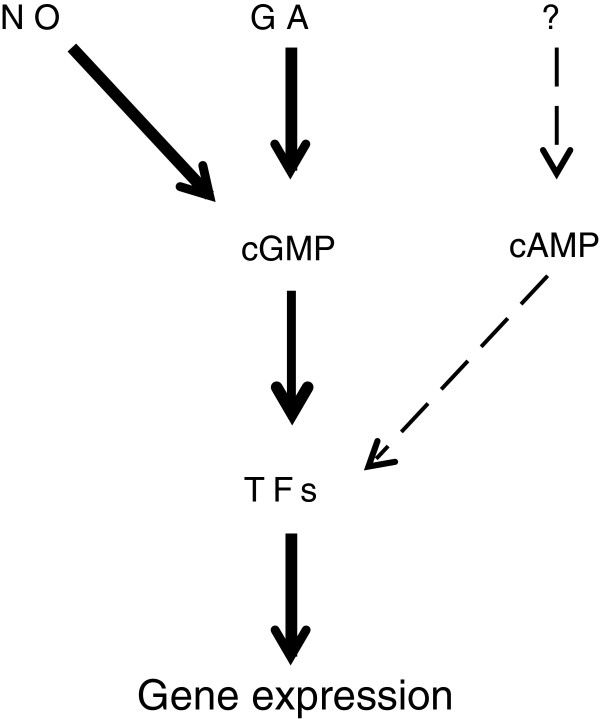
**Schematic diagram showing the relationship between cGMP, the plant hormone giberellic acid (GA), nitric oxide (NO) and induction of gene expression in plant cells.** Both GA and NO can induce increases in cGMP levels which in turn stimulate specific transcription factors (TFs) to induce gene transcription. The interaction of cAMP with cGMP is less well understood in plants so the induction of cAMP production is indicated by a question mark (?) and dashed lines.

Cyclic AMP (cAMP) and cyclic GMP (cGMP) are major signaling molecules generated from ATP or GTP by the action of adenylate cyclases or guanylate cyclases, respectively. Cyclic AMP is found in all organisms where it regulates enzyme activity and transcription factors although its role(s) in plants is poorly documented possibly because cAMP levels in plants are considerably lower than in vertebrates and there is a lack of annotated adenylate cyclases [1-3]. In mammalian cells cGMP is a transitory molecule that directly regulates cyclic nucleotide gated ion channels, protein kinases and activates specific phosphodiesterases that degrade cGMP [4]. In mammalian systems the physiological roles of cGMP are well characterized in intestinal fluid and electrolyte homeostasis, phototransduction, and vascular smooth muscle where it mediates relaxation [4]. Guanylate cyclases and cGMP are well represented in various invertebrates such as insects, nematodes and echindermata; and the amoeba Dictyostelium also uses cGMP as a chemo-attractant [5,6]. The role of cGMP in bacteria, fungi and plants has been controversial [1,6-9]. However cGMP is now a relatively well characterized second messenger in higher plants (Figure 1) mediating a wide variety of physiological effects ranging from plant hormone dependent responses to induction of plant defense responses [7,9] and novel guanylate cyclases have been partly characterized [10-15]. Although a guanylate cyclase from the cyanobacteria has been crystallized [16] it is only recently that a guanylate cyclase and cGMP system involved in bacterial encystment has been revealed in *Rhodospirillum centenum* and by homology in other members of the α-proteabacteria such as *Rhizobium NGR234*[17]. Therefore cGMP appears to be a universal signaling molecule in eukaryotic cells and to have roles in at least some prokaryotes.

Part of the controversy relating to cGMP being a second messenger in non-animal cells is due to its transitory nature and generally lower levels compared to cAMP. The most reliable method to detect cGMP is by mass spectrometry [7,9] which is limited in its utility for time course measurements essential for detecting molecules transiently present in limited numbers of cells. Various antibody based assays have been developed but these were tailored for animal cells and the early assays detected spurious components in non-animal cells [9]. Newer ELISA based kits are more specific for cGMP but tend to under report amounts of cGMP present in non-animal cells [13]. In plant cells where there is a large vacuole, it is feasible that local concentrations of cGMP are much higher than detected with either mass spectrometry or antibody based kits. To measure endogenous cGMP levels at a cellular level in real time in mammalian cells a fluorescent biosensor FlincG was developed. FlincG contains the regulatory domain of protein kinase G type I fused to the circular permuted enhanced green fluorescent protein (GFP) and has been used in transfected mammalian cells to detect intracellular changes in cGMP over native dynamic ranges [18]. Constructs of FlincG placed under the control of a plant promoter have been expressed in plant cells and detect endogenous changes in cGMP levels [19,20]. Alternative protein cGMP biosensors have been constructed using cGMP binding domains in phosphodiesterases (e.g. PDE5) and the blue fluorescent protein (mTagBFP) that have been used in multiple parameter imaging with FRET-based cAMP reporters in animal cells [21]. Promoter reporter constructs containing the cAMP binding protein response element have been extensively used in mammalian systems to detect changes in cAMP that alter gene expression [22-25]. Several luciferase based reporter systems augmented with cAMP binding protein response element are commercially available. Significant cross-talk occurs between the cAMP and cGMP transcription regulatory pathways in mammalian cells [4] and hence these reporter systems have also been used to detect changes in gene expression via cGMP in mammalian cells [26,27]. The complexities of the interaction between the cyclic nucleotide pathways has led to the development of a mammalian cGMP reporter system pathways using the cAMP response element and overexpressed protein kinase G but this system is unable to discriminate between cAMP and cGMP [28]. Despite these advances in mammalian cells, a reporter assay for plant cells that detects gene expression induced by changes in intracellular levels of either cGMP or cAMP levels is lacking.

Exogenous application of membrane permeable cGMP in plant cells has been shown to alter the transcriptome [29]. Maathuis [29] identified several genes that were up-regulated in root cells following exposure to membrane permeable cGMP. We selected three of these genes as being potentially suitable candidates to develop promoter:luciferase reporter constructs that result in the downstream induction of gene expression in plant cells following intracellular changes in cGMP levels. We show changes in expression of luciferase following treatment of plant cells with membrane permeable cyclic nucleotides with one of these constructs. Since this candidate promoter contained a cGMP response element identified in mammalian cells and another that was associated with gibberellic acid (GA), we augmented the promoter with these response elements and tested them in transiently transfected plant cells. In addition, we tested the effectiveness of the candidate promoters on bacterial cells where we observed that they reported changes in gene expression following treatments where cyclic nucleotides were applied exogenously or generated endogenously.

## Results and discussion

### Generation of cyclic nucleotide responsive promoter:luciferase plasmid constructs

**Figure 2:**
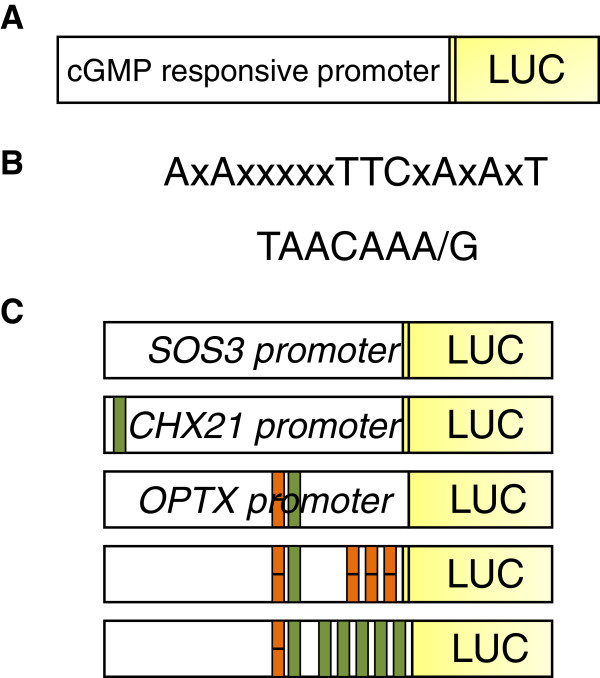
**Schematic diagrams of plasmids used and the response elements incorporated. (A)** cGMP responsive promoter LUC plasmid used for transfection. **(B)** Mammalian cGMP response element (cGMPRE) DNA sequence described in Hum *et al*. [22] top and gibberellic acid (GA) response element (GARE) DNA sequence described in Bastian *et al*. [35] bottom. **(C)** Schematic diagram of plasmids used for transfection. SOS3 promoter; CHX21 promoter showing the GARE present (green); OPTX promoter showing the mammalian cGMPRE (orange, horizontal stripe) and GARE (green) present, cGMPRE inserted and GARE inserted.

We selected three *Arabidopsis thaliana* genes previously shown by Maathuis [29] to be induced by membrane permeable cGMP (8-bromoguanosine 3′,5′-cyclic monophosphate sodium salt or 8-bromo cGMP) in root tissue. *SALT OVERLY SENSITIVE 3 (SOS3)*, *CATION/H*^*+*^*EXCHANGER 21 (CHX21)* and *OLIGOPEPTIDE TRANSPORTER X (OPTX)* all showed at least a two fold increase in expression that was verified by quantitative RT-PCR [29]. The *A. thaliana OPTX* gene has been annotated as a member of the plant oligopeptide transporter family (although it was originally annotated as *NTL1* (*LOW AFFINITY NITRATE TRANSPORTER*)) and is predicted to be a membrane bound transporter of small peptides. Promoter fragments of approximately 1000 bp of *SOS3*, *CHX21* and *OPTX* were amplified and cloned into the plant luciferase vector pLucTrap3(GW) [30] to create p*SOS3*:*LUC*, p*CHX21*:*LUC* and p*OPTX*:*LUC* (Figure 2A and 2C).

**Figure 3:**
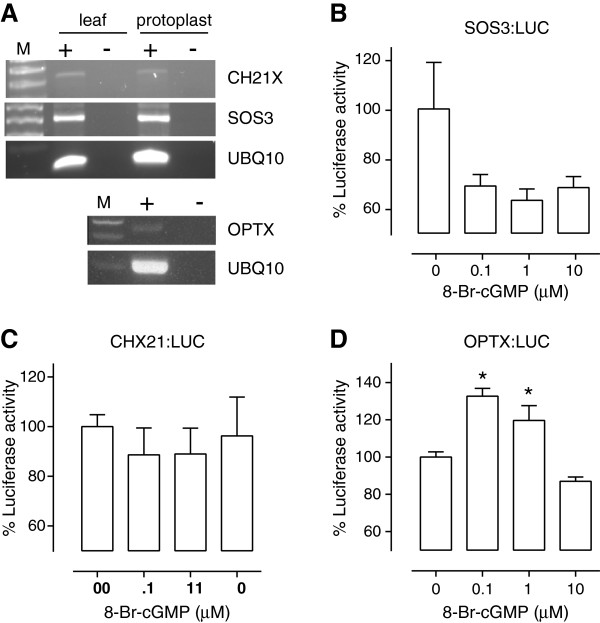
**Effect of cGMP on the luciferase reporter constructs in plant protoplasts. (A)** Detection of CHX21, SOS3 and OPTX transcripts in Arabidopsis leaf mesophyll protoplasts. Control amplifications were also made of the UBQ10 gene from RNA samples with (+, plus) or without (−, minus) reverse transcriptase (RT) for protoplasts (OPTX, SOS3 and CHX21) or leaf tissue (SOS3 and CHX21 only) and M refers to the lane containing the DNA size ladder. **(B-D)** Arabidopsis leaf mesophyll protoplasts were transiently transfected with a promoter luciferase reporter construct (**B**: SOS3:LUC (n = 4 – 5); **C**: CHX21:LUC (n = 3 – 4); **D**: OPTX:LUC (n = 3–6)) and treated with 0, 0.1, 1 or 10 μM 8-bromo cGMP. At least 3 biological replicates were completed on different days for each promoter treatment combination. Luciferase was normalized against protein content and asterisks indicate treatments significantly different from the control (P < 0.05; one way ANOVA, Dunnett’s multiple comparison post-test).

**Figure 4:**
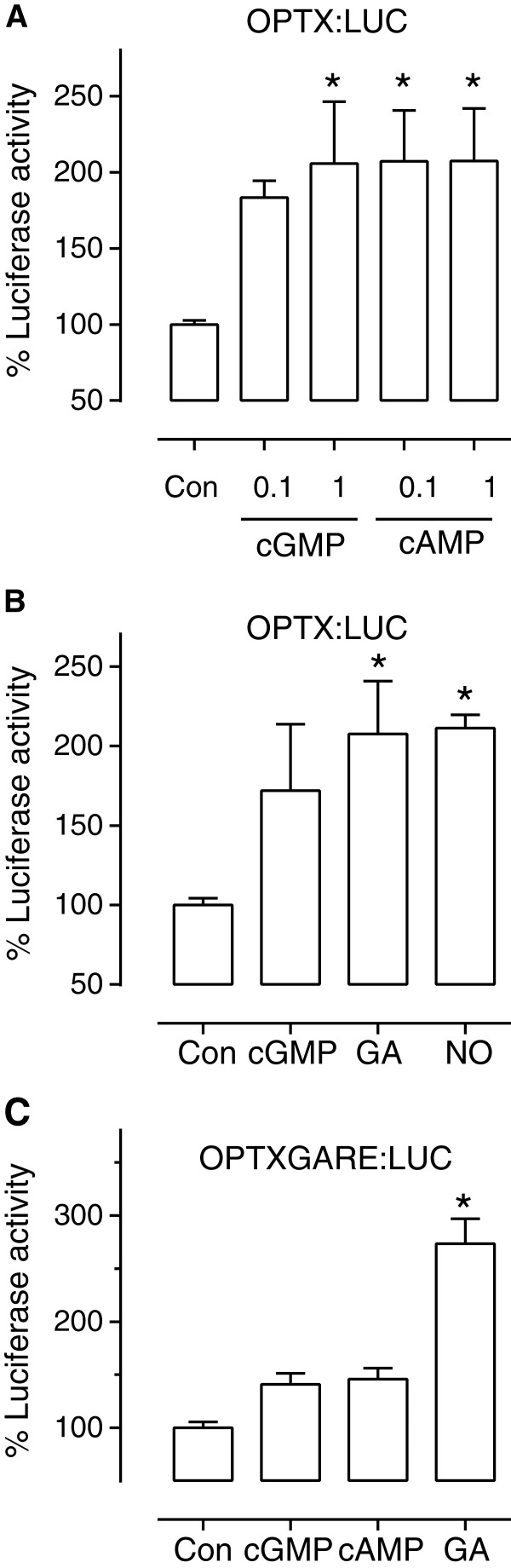
**Cyclic nucleotide induced luciferase activity in plant protoplasts.** Arabidopsis leaf mesophyll protoplasts were transiently transfected with a promoter luciferase reporter construct and treated as described below. At least 3 biological replicates were completed on different days for each promoter treatment combination. **(A)** OPTX:LUC treated with 0.1 or 1 μM 8-bromo cGMP or dibutyryl cAMP (n = 8–9); **(B)** OPTX:LUC treated with 0.1 μM 8-bromo cGMP, 30 μM GA or 30 nM DEA/NONOate (n = 6); **(C)** OPTXGARE:LUC treated with 0.1 μM 8-bromo cGMP, 0.1 μM dibutyryl cAMP or 30 μM GA (n = 3). Luciferase was normalized against protein content and asterisks indicate treatments significantly different from the control (P < 0.05; one way ANOVA, Dunnett’s multiple comparison post-test).

We determined that SOS3, CHX21 and OPTX were all expressed in freshly isolated leaf mesophyll protoplasts (Figure 3A) despite these genes being originally identified in root tissue [29]. Analysis of Arabidopsis microarray data through Genevestigator [31] also indicates that these genes are all expressed in leaf tissue. Hence the machinery (i.e. transcription factors and other signaling molecules) that control their expression is likely to be present in leaf tissue as well as roots. We then tested the luciferase activity of each promoter in freshly prepared Arabidopsis mesophyll protoplasts treated with membrane permeable 8-bromo cGMP at various concentrations (0, 0.1, 1, and 10 μM). Luciferase activity was quantified and standardized by protein content for each sample and results were expressed as percentage of luciferase activity where the untreated (no cGMP treatment) is equal to 100%. There was no significant difference in luciferase activity for the *SOS3* or *CHX21* promoters at any cGMP concentration (Figure 3B and 3C). However, the *OPTX* promoter showed a significant increase in luciferase activity with 8-bromo cGMP treatments at 0.1 and 1 μM (Figure 3D). The specificity of the promoter luciferase *OPTX:LUC* construct was tested by treating protoplasts with a membrane permeable form of cAMP, N6,2’-O-dibutyryladenosine 3’:5’-cyclic monophosphate (or dibutyryl cAMP). Dibutyryl cAMP and 8-bromo cGMP induced similar levels of luciferase activity in protoplasts transiently transfected with *OPTX:LUC* (Figure 4A) indicating that both cyclic nucleotides can up-regulate expression of the *OPTX* gene. Whether this is because response elements in the promoter are not specific for cGMP in plants or additional cAMP response elements are present remains to be determined. It is possible that like mammalian cells [4] there is considerable cross talk between the machinery controlling gene expression in plant cells in response to cyclic nucleotides. However, of the three promoters examined in this study, only the OPTX promoter contains both a mammalian cGMP and a putative GA response element (Figure 2C).

Nitric oxide has been shown to lead to increases in cytosolic cGMP levels in plants [32,33] as well as animal systems [18,34]. To test whether the promoter luciferase construct *OPTX:LUC* could be used to show physiological differences in cGMP levels within plant protoplasts we used the nitric oxide donor DEA/NONOate at 30 nM which has been shown to induce increases in cytosolic cGMP in plants [19]. The plant hormone gibberellic acid (GA) has also been shown to raise cGMP levels in plants and induce gene expression at concentrations such as 30 μM [19,20,35,36]. Therefore we used a 30 μM GA treatment as well in the transiently transfected protoplast experiments. Protoplasts transfected with *OPTX:LUC* showed a significant increase in luciferase activity when compared to the untreated control for both the 30 μM GA and 30 nM DEA/NONOate treatments (Figure 4B).

### Augmentation of the cyclic nucleotide sensitive promoter *OPTX*

DNA sequence analysis of the three promoters showed that only the *OPTX* promoter contained the mammalian cGMP response element (AxAxxxxxTTCxAxAxT; -361 bp upstream from ATG; Additional file 1) identified by Hum *et al*. [22] (Figure 2B and 2C). To our knowledge this cGMP response element is the only element responding to changes in intracellular cGMP that has been characterized in animal cells or plant cells. To potentially enhance cGMP sensitivity of the *OPTX* promoter we used mutagenesis to incorporate an additional three mammalian cGMP response elements to make *OPTXcGMPRE:LUC* (Figure 2C). Arabidopsis protoplasts transfected with the *OPTXcGMPRE*:*LUC* showed no significant difference in luciferase activity for any of the cGMP concentrations tested when compared to the untreated control (data not shown). This could be because the mammalian response element is ineffective in plants or alternatively it may be due to the positioning of the augmented response elements close to the transcription start site where cGMP activated DNA binding proteins may not correctly modulate the transcriptional machinery as the cGMP response element is over 1000 bp upstream of NPR1/GCA in the mammalian system [22,37].

A promoter analysis study identified a putative GA response element (TAACAAA/G; Figure 2B) which is found at a higher frequency in promoters of genes responsive to GA [35]. Both the *OPTX* and *CHX21* promoters contained a GA response element sequence. The GA response element is located −194 bp upstream from ATG in the OPTX promoter (Additional file 1) and is found much further from the start of the gene in *CHX21* (−1023 bp upstream from ATG; Additional file 1). Since GA induces transient increases in cGMP that in turn directly alters expression of GA-induced genes in plants [36], we used mutagenesis to incorporate five additional GA response elements into the *OPTX* promoter to make *OPTXGARE:LUC* (Figure 2C). Both the membrane permeable analogues of cGMP and cAMP increased luciferase activity to similar levels in protoplasts transfected with *OPTXGARE:LUC* (Figure 4C) which limits using this construct to reporting gene induction by either cyclic nucleotide. Importantly, GA treatment also significantly increased luciferase activity (Figure 4C) supporting the premise that the response element is likely to be activated downstream of increases of cyclic nucleotides such as cGMP induced by GA (Figure 1). These findings demonstrate that the OPTX and OPTXGARE promoter reporter systems can be used to report gene induction following increases in cyclic nucleotide levels thereby reporting on a complete pathway mediated through changes in cyclic nucleotide levels as distinct from reporting specific changes in levels of the cyclic nucleotides. When employed together with studies using FlincG that reports on intracellular transient increases of cGMP [19,20], the two systems will allow unraveling of conditions and levels of cyclic nucleotides necessary to lead to changes in gene transcription.

### *OPTX* promoters report cyclic nucleotide induced gene activity in bacterial cells

**Figure 5:**
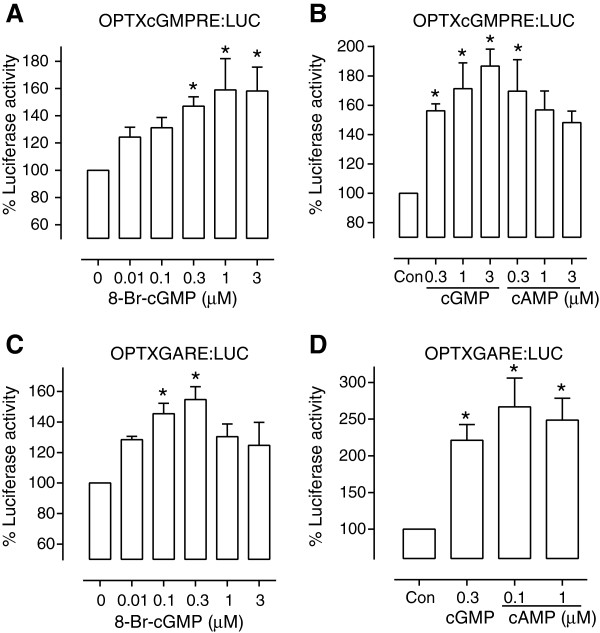
**Cyclic nucleotide induced luciferase activity in *E. coli.*** BL21-AI cells transformed with a promoter luciferase reporter construct and treated with different concentrations of 8-bromo cGMP and dibutyryl cAMP. At least 3 separate colonies were tested for each promoter / treatment combination. **(A)** Effect of different concentrations of 8-bromo cGMP on B21A-AI cells transformed with OPTXcGMPRE:LUC (n = 3–4); **(B)** Effect of dibutyryl cAMP on B21A-AI cells transformed with OPTXcGMPRE:LUC (n = 3–4); **(C)** Effect of different concentrations of 8-bromo cGMP on B21A-AI cells transformed with OPTX-GARE:LUC (n = 4). **(D)** Effect of dibutyryl cAMP on B21A-AI cells transformed with OPTXGARE:LUC (n = 6). Luciferase activity was expressed relative to OD_600_ to normalize results per cell number and asterisks indicate treatments significantly different from the control (P < 0.05; one way ANOVA, Dunnett’s multiple comparison post-test).

To date, only members of the α-proteabacteria are known to naturally synthesize cGMP although other bacteria produce cAMP and the di-cyclic nucleotides c-di-AMP and c-di-GMP [1,17]. Therefore a system that detects cGMP in bacteria would be particularly useful to screen for novel and functional recombinant guanylate cyclases from other organisms. One of our goals is to develop an assay that we can use to detect novel recombinant guanylate cyclases expressed in bacteria. With this concept in mind, we tested the three OPTX constructs in the BL21-AI *E. coli* strain which we selected as a representative bacterium because it is suitable for high-level recombinant protein expression. The *OPTX:LUC*, *OPTXcGMPRE:LUC* and *OPTXGARE:LUC* were transformed separately into the BL21-AI *E. coli*. At least four independently transformed colonies were grown and tested for each promoter/ treatment combination. We normalized each sample using the OD_600_ reflecting cell number for the sample and expressed the luciferase activity as a percentage of the untreated control which was set at 100%. The promoter *OPTX:LUC* showed no significant difference in luciferase activity from the untreated control for 0.01 - 3 μM cGMP treatments (data not shown). Conversely, expression of both the augmented constructs was significantly induced by cyclic nucleotide treatments. The *OPTXcGMPRE:LUC* transformants showed a significant increase in luciferase activity when treated with 0.3 μM, 1 μM and 3 μM 8-bromo cGMP but not 0.01 μM and 0.1 μM 8-bromo cGMP (Figure 5A). However, this increase in luciferase activity appears to be independent of the purine base as similar changes were seen with treatments containing dibutyryl cAMP (Figure 5B). The *OPTXGARE:LUC* showed a significant increase in luciferase activity when compared to the untreated control when 8-bromo cGMP was between 0.1 μM and 0.3 μM but not at 0.01 μM or 1 μM (Figure 5C). Again similar increases were observed in response to dibutyryl cAMP in BL21-AI cells transformed with *OPTXGARE:LUC* (Figure 5D). To date no known guanylate cyclases or cGMP signaling system has been identified in *E. coli* although highly characterized cAMP signal networks are present [1]. One possibility is that the native bacterial transcription factors recognize both purine cyclic nucleotides without discriminating between them although it needs to be shown that *E. coli* transcription factors can bind to response elements in plant promoters.

**Figure 6:**
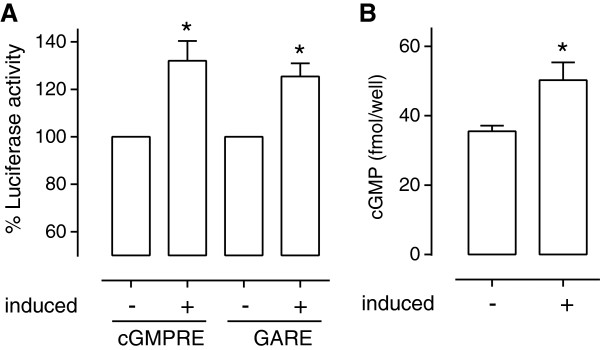
**Detection of cGMP induced by the novel guanylate cyclase PSKR1 in bacteria. (A)** BL21-AI cells were co-transformed with the specified promoter luciferase reporter construct (OPTXcGMPRE:LUC or OPTXGARE:LUC; n = 4) and a plasmid containing a novel guanylate cyclase enzyme (pDESTPSKR1cd). Expression of PSKR1 was induced (+) in at least 4 separate colonies and then luciferase activity was tested and expressed relative to culture optical density to normalize results per cell number and reported relative to the non-induced control (−) co-transfected bacteria grown under the same conditions. **(B)** Relative amount of cGMP detected in non-induced (−) and induced (+) bacteria co-transfected with PSKR1 and luciferase reporter constructs. Asterisks indicate treatments significantly different from control non-induced treatments (P < 0.05; **A**: one way ANOVA, Tukey-Kramer multiple comparison post-test or **B**: student’s un-paired t-test).

**Figure 7:**
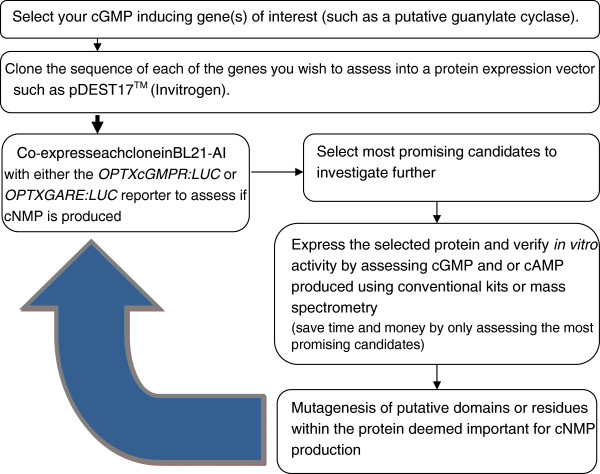
**Flow chart showing a sample experimental plan.** The flow of experiments show that by assessing the putative proteins of interest using the *OPTXcGMPRE:LUC* or alternatively the *OPTXGARE:LUC* reporter the researcher can determine the ability of each recombinant protein to induce cGMP or cAMP *in vivo*. The most promising candidates can be selected for further analysis as recombinant proteins using conventional kits. Once the proteins are functionally verified, mutagenesis of putative functional domains or residues can be made then the researcher can again utilize the *OPTXcGMPRE:LUC* or alternatively the *OPTXGARE:LUC* reporter system to determine the most promising candidates to pursue.

The results shown in Figure 5 encouraged us to test if we could detect cGMP in BL21-AI cells expressing a novel guanylate cyclase. Previous work using BL21 cells expressing the novel plant cytoplasmic guanylate cyclase GC1 indicated that cGMP is generated within bacteria during the period when protein expression is being induced [12,15]. We have demonstrated that the cytoplasmic domain of the plant phytosulfokine receptor (PSKR1) contains guanylate cyclase but not adenylate cyclase activity [11] and chose this as the test protein. We co-transformed bacteria with the cytoplasmic domain of PSKR1 and either *OPTXcGMPRE* or *OPTXGARE* to test if the expressed PSKR1 generates cGMP that in turn induces downstream luciferase activity. At 3 hours following induction of PSKR1 expression, the luciferase activity is increased relative to the un-induced control treatments indicating that this is indeed the case (Figure 6A). The significant albeit small increase in cGMP levels in the induced compared to the un-induced control bacteria (Figure 6B) correlates with the induction of luciferase activity. This is an exciting finding as it indicates that these bacterial reporter assays can be used as a preliminary functional screen for novel guanylate cyclase enzymes. The advantage of such a preliminary screen is that recombinant proteins containing guanylate cyclase activity are identified prior to time consuming full characterization studies as outlined in Figure 7.

## Conclusions

We have developed promoter reporter systems based on the plant *OPTX* promoter that can be employed in bacteria and plant cells to report changes in gene expression reflecting alterations in endogenous cyclic nucleotide levels. The promoter is from the plant gene *OPTX* and augmentation of the promoter reporter with cGMP and GA response elements resulted in changes of efficacy in the different cell types. In plant cells the *OPTX* and GARE augmented reporter do not discriminate between cGMP and cAMP but both respond to changes in intracellular cGMP levels and both can be readily used in transiently transfected protoplasts. Augmentation of the OPTX promoter with either the GA or the separate cGMP response elements allowed detection of increased levels of cyclic nucleotides in bacteria. The augmented promoters can also be employed to detect the activity of novel recombinant guanylate cyclases in bacteria (Figure 7).

## Methods

### Plasmid construction

The promoter sequences for *OLIGOPEPTIDE TRANSPORTER X* (*OPTX*) [TAIR:AT1G33440; GenBank:NM_103069.3], *SALT OVERLY SENSITIVE 3* (*SOS3*) [TAIR:AT5G24270; GenBank:NM_122333] and *CATION/H*^*+*^*EXCHANGER 21* (*CHX21*) [TAIR:AT2G31910; GenBank:NM_128749.2] were amplified from freshly prepared *Arabidopsis thaliana* (Columbia-0) wild type genomic DNA using primer pairs OPTX-P-fwd with OPTX -P-rev, CHX21-P-fwd with CHX21-P-rev, and SOS3-P-fwd with SOS3-P-rev, respectively (see Additional file 2: Table S1 for primer sequences). These PCR products were used as templates to incorporate gateway recombination sites resulting in ~1000 bp promoter fragments with the respective ATG start codon of each gene. The PCR products were recombined separately into pDONR207 and confirmed by sequencing before recombination into the p*LUCTrap3* [GenBank:AY968054.1] [30] resulting in p*OPTXLUC*, p*CHX21LUC* and p*SOS3LUC* (see Additional file 1 for full sequence). Sequencing confirmed the *LUCIFERASE* encoding sequence was in frame with the respective promoters. Three copies of the cGMP Response Element (RE) [22] and five copies of the Gibberellic Acid RE (GARE) [35] were incorporated into the *A. thaliana OPTX* promoter using a Phusion site-directed mutagenesis kit (Finnzymes, Thermo Scientific, Scoresby VIC, Australia) following the manufacturer’s instructions. The *OPTX* promoter in p*DONR207* was used as the template with either OPTX-GARE-fwd or OPTX-cGMPRE-fwd and OPTX-RE-rev primer pairs. Sequence was confirmed before the respective promoter fragments were recombined into p*LUCTrap3* to make p*OPTXcGMPRELUC* and p*OPTXGARELUC* (see Additional file 1 for full sequences).

### Plant growth, protoplast preparation and treatments

*Arabidopsis thaliana* (Col-0) leaves from 5 to 6 week old plants were used to prepare protoplasts as previously described [38] and genomic DNA and total RNA using Qiagen DNeasy and RNeasy plant kits (Qiagen, Melbourne VIC, Australia) following the manufacturer’s instructions except total RNA was treated with Ambion DNase (Life Technologies, Melbourne VIC, Australia). PureLink HiPure plasmid filter purification kit (Life Technologies) was used to purify transfection plasmid and for each sample, 2 x 10^5^ protoplasts were transfected with 100 μg p(promoter)*LUCTRAP3(OPTX, CHX21, SOS3, OPTXcGMPRE* or *OPTXGARE*) and 900 ng of p*UBQ10:GUS* as a comparative transfection control. Protoplasts recovered in the dark at room temperature for 2 h before treatment for 3 or 18 h with various concentrations of 8-bromo cGMP (Sigma, Castle Hill NSW, Australia), N6,2’-O-dibutyryladenosine 3’:5’-cyclic monophosphate (dibutyryl cAMP; Sigma), DEA/NONOate (Sigma) or gibberellic acid (Sigma). Preliminary results indicated that only low levels of luciferase were detected after 3 hour treatments as we have previously observed [39] so 18 hour treatments were used for the promoter analysis reported here as this is a common time point used in luciferase reporter assays [40]. Following treatment each protoplast sample was resuspended in 100 μl of 1X Reporter Lysis Buffer (RBL, Promega, Sydney Australia) vortexed, centrifuged, then lysate was frozen in liquid nitrogen and stored at −80°C. cDNA was generated using 100 ng of total RNA, oligodT primer and SuperScript III Reverse Transcriptase (Life Technologies). For each RNA template two identical reactions either with (+) or without (−) SuperScript III Reverse Transcriptase where included to ensure PCR products were amplified from the cDNA generated and not contaminating genomic DNA. Volumes of 1 (UBQ10) to 4 μl (SOS3, CHX21, OPTX) from cDNA reactions were used as template in PCR reactions and primers are described in additional material (see Additional file 2: Table S1).

### Bacterial treatments

Competent BL21-AI cells (Life Technologies) were separately transformed with p*LUCTRAP3(OPTX, OPTXcGMPRE* or *OPTXGARE).* At least four isolated colonies for each plasmid were separately inoculated into 10 ml LB broth with kanamycin (50 μg ml^-1^) and grown overnight. One ml of overnight culture was used to inoculate 10 ml LB broth with antibiotic selection and incubated at 37°C in a shaking incubator to anOD_600_ of 0.5. Then 1 ml aliquots of each culture were treated with specific concentrations of 8-bromo cGMP or dibutyryl cAMP for 3 hours with shaking in 1.5 ml tubes. The OD_600_ of each culture was measured to quantify the cell number using a UV/VIS spectrophotometer (Spectum). After the 3 hour treatment, a 90 μl bacterial sample was mixed with 10 μL of buffer (1 M K_2_HPO_4_ at pH 7.8 and 20 mM EDTA) and the cultures were frozen on dry ice. In some cases, BL21-AI cells were co-transformed with either *OPTXcGMPRE* or *OPTXGARE* in p*LUCTRAP3* (kanamycin resistance) and the cytoplasmic domain of the phytosulfokine receptor 1 (PSKR1) [TAIR:AT2G02220; GenBank:NP_1783300.1] in p*DEST17* (p*DEST17PSKR1cd*; ampicillin/carbenicillin resistance) [11]. Colonies were selected with kanamycin (100 μg ml^-1^) and carbenicillin (200 μg ml^-1^), separately inoculated into LB broth containing both antibiotics and grown to OD_600_ of about 0.5 when the BL21-AI cells were induced with 0.2% L-arabinose and 1 mM IPTG to express the PSKR1 protein for 3 hours. Triplicate technical replicates of at least three independent co-transfected sample lysates were assessed using the Amersham cGMP enzyme immunoassay Biotrak (EIA) system following the standard (Protocol 3) as described in the supplier’s manual (code RPN226, GE Healthcare, Rydalmere NSW, Australia). The results were correlated with cell number and expressed relative to the un-induced sample as different colonies produced different amounts of cGMP ranging from 30 to 200 fmol per sample.

### Luciferase assay

Protoplasts and bacteria luciferase activity was assessed using Promega’s Luciferase assay system (Promega, Sydney NSW, Australia) following the manufacturer’s instructions. Luminescence of triplicate technical replicates on a white 96 well plate was measured with the enhanced luminescence option using an EnVision 2101 plate reader (PerkinElmer, Melbourne VIC, Australia). For bacteria, luciferase activity was expressed relative to OD_600_ to normalize results per cell number. For protoplasts, luciferase was initially normalized against β-glucuronidase (GUS) activity as described previously [39]. However comparison of luciferase activity normalization using β-glucuronidase versus protein revealed no significant difference as we have previously observed [39] hence protoplast protein amount was used for normalization in the data reported here. Protein was assessed using a Nanodrop (Thermofisher Scientific, Scoresby VIC, Australia).

### Statistical analyses

Data was analyzed using one way analysis of variance (ANOVA) followed by either Dunnett’s multiple comparison test (P < 0.05) or Tukey-Kramer multiple comparison test (P < 0.05) using GraphPad Prism 6.0 (GraphPad Software, La Jolla CA, USA). Where only two treatments were compared, data was analyzed using the un-paired student’s t-test. Data for each treatment contains at least 3 biological replicates (n ≥ 3) and each experiment was repeated at least twice.

## Abbreviations

CHX21: CATION/H^+^ EXCHANGER 21; GA: Giberellic acid; LUC: Luciferase; NPR1/GCA: Natriuretic peptide receptor A / guanylate cyclase A; OPTX: Oligopeptide transporter X; OPTXcGMPRE: OPTX promoter augmented with mammalian cGMP response element; OPTXGARE: OPTX promoter augmented with GA response element; SOS3: SALT OVERLY SENSITIVE 3.

## Competing interests

The authors declare that they have no competing interests.

## Authors’ contributions

HRI and JIW conceived the idea, all authors designed the experiments, JIW and LF carried out the experimental work, HRI wrote the manuscript with critical input from JIW and LF. All authors read and approved the final manuscript.

## Supplementary Material

Additional file 1 The file contains the text sequences of pSOS3:LUC, pCHX21:LUC, pOPTX:LUC, pOPTXcGMPRE:LUC and pOPTXGARE:LUC used in this study. Click here for file

Additional file 2: Table S1 Primer sequences used in this study. Click here for file
