# Supplementary material for: A cyclic nucleotide sensitive promoter reporter system suitable for bacteria and plant cells
Source: BMC Biotechnol. 2013 Nov 9;13:97. doi: 10.1186/1472-6750-13-97 (PMC3829209; doi:10.1186/1472-6750-13-97)
Supplement: Additional file 1 — The file contains the text sequences of pSOS3:LUC, pCHX21:LUC, pOPTX:LUC, pOPTXcGMPRE:LUC and pOPTXGARE:LUC used in this study. [file 1472-6750-13-97-S1.docx]

**Additional file 1:** The file contains the text sequences of pSOS3:LUC, pCHX21:LUC, pOPTX:LUC, pOPTXcGMPRE:LUC and pOPTXGARE:LUC used in this study.

>pSOS3:LUC

agcccgctcgacggcgggctgttggatggggatcgcctgaatcgccccatcatccagcca

gaaagtgagggagccacggttgatgagagctttgttgtaggtggaccagttggtgatttt

gaacttttgctttgccacggaacggtctgcgttgtcgggaagatgcgtgatctgatcctt

caactcagcaaaagttcgatttattcaacaaagccacgttgtgtctcaaaatctctgatg

ttacattgcacaagataaaaatatatcatcatgaacaataaaactgtctgcttacataaa

cagtaatacaaggggtgttatgagccatattcaacgggaaacgtcttgctccaggccgcg

attaaattccaacatggatgctgatttatatgggtataaatgggctcgcgataatgtcgg

gcaatcaggtgcgacaatctatggattgtatgggaagcccgatgcgccagagttgtttct

gaaacatggcaaaggtagcgttgccaatgatgttacagatgagatggtcagactaaactg

gctgacggaatttatgcctcttccgaccatcaagcattttatccgtactcctgatgatgc

atggttactcaccactgcgatcccagggaaaacagcattccaggtattagaagaatatcc

tgattcaggtgaaaatattgttgatgcgctggcagtgttcctgcgccggttgcattcgat

tcctgtttgtaattgtccttttaacagcgatcgcgtatttcgtctcgctcaggcgcaatc

acgaatgaataacggtttggttgatgcgagtgattttgatgacgagcgtaatggctggcc

tgttgaacaagtctggaaagaaatgcataaacttttgccattctcaccggattcagtcgt

cactcatggtgatttctcacttgataaccttatttttgacgaggggaaattaataggttg

tattgatgttggacgagtcggaatcgcagaccgataccaggatcttgccatcctatggaa

ctgcctcggtgagttttctccttcattacagaaacggctttttcaaaaatatggtattga

taatcctgatatgaataaattgcagtttcatttgatgctcgatgagtttttctaatcaga

attggttaattggttgtaaccatgaccaaaatcccttaacgtgagttttcgttccactga

gcgtcagaccccgtagaaaagatcaaaggatcttcttgagatcctttttttctgcgcgta

atctgctgcttgcaaacaaaaaaaccaccgctaccagcggtggtttgtttgccggatcaa

gagctaccaactctttttccgaaggtaactggcttcagcagagcgcagataccaaatact

gtccttctagtgtagccgtagttaggccaccacttcaagaactctgtagcaccgcctaca

tacctcgctctgctaatcctgttaccagtggctgctgccagtggcgataagtcgtgtctt

accgggttggactcaagacgatagttaccggataaggcgcagcggtcgggctgaacgggg

ggttcgtgcacacagcccagcttggagcgaacgacctacaccgaactgagatacctacag

cgtgagctatgagaaagcgccacgcttcccgaagggagaaaggcggacaggtatccggta

agcggcagggtcggaacaggagagcgcacgagggagcttccagggggaaacgcctggtat

ctttatagtcctgtcgggtttcgccacctctgacttgagcgtcgatttttgtgatgctcg

tcaggggggcggagcctatggaaaaacgccagcaacgcggcctttttacggttcctggcc

ttttgctggccttttgctcacatgagatctcaaacaaacacatacagcgacttagtttac

ccgccaatatatcctgtcaaggcctatcaacaagtttgtacaaaaaagcaggctagattt

gatattgatccggtatttgtatttatgtattatctatatgtatgcatatatcataggaga

ctttgatcatttatctatgttattagggataataaaattaggagtaaactaattatctct

tatatctatacacctagtcattagcgtggctaatacggcgacttgcctacaccttctgtg

aattatataatatttggtttcattacgtttattgtgaatcattactaaattatagattaa

atagggaaaacgctgtttttgttctaattttggctacttctgttaatgaattgattgttt

agggagagcttactagctgtcgtgaaagctacaactattttaatggctaactttgccacc

tatatcctatgtatttattatgtaaaaaaaaaaatgtataaaccaatgacataatcgttc

gtatagccgtcgacttctattgtatatttgctcaaacgtcgaccctttcaaaattttaat

atcgatgtttgtcaaactcttgcagattttgatgaattcgacgctagattgcctttaacg

aaattcaaatgttacatatacatagacgatatatatatacatgcattagatgtacatgat

tagtgctcatctacattaatatttcatgtatgtatatgtaattaaatattgaatttcatt

agaaacattttggtaatctcgtgagtttgccaaaatatgtttactttgtttgagtttgac

cacaaaaaaaacactagaaaagtcaataacgtgacaaactaaacaagttgcctattatta

attgcactcgatcttaattggtttgttctaggaccatttttagtagaagtaaaaccagcg

aaaacgaatgtaggattcagtcgaagggtgttttataaaatcatcaaattattaggaaca

atgaaatatctgtaaattaaccaagattatttgcgtttggttgtagcgtgagaaaagcaa

aggctaataacagttgagagaagggtgtgtttgtATGgacccagctttcttgtacaaagt

ggttgccatggaagacgccaaaaacataaagaaaggcccggcgccattctatccgctgga

agatggaaccgctggagagcaactgcataaggctatgaagagatacgccctggttcctgg

aacaattgcttttacagatgcacatatcgaggtggacatcacttacgctgagtacttcga

aatgtccgttcggttggcagaagctatgaaacgatatgggctgaatacaaatcacagaat

cgtcgtatgcagtgaaaactctcttcaattctttatgccggtgttgggcgcgttatttat

cggagttgcagttgcgcccgcgaacgacatttataatgaacgtgaattgctcaacagtat

gggcatttcgcagcctaccgtggtgttcgtttccaaaaaggggttgcaaaaaattttgaa

cgtgcaaaaaaagctcccaatcatccaaaaaattattatcatggattctaaaacggatta

ccagggatttcagtcgatgtacacgttcgtcacatctcatctacctcccggttttaatga

atacgattttgtgccagagtccttcgatagggacaagacaattgcactgatcatgaactc

ctctggatctactggtctgcctaaaggtgtcgctctgcctcatagaactgcctgcgtgag

attctcgcatgccagagatcctatttttggcaatcaaatcattccggatactgcgatttt

aagtgttgttccattccatcacggttttggaatgtttactacactcggatatttgatatg

tggatttcgagtcgtcttaatgtatagatttgaagaagagctgtttctgaggagccttca

ggattacaagattcaaagtgcgctgctggtgccaaccctattctccttcttcgccaaaag

cactctgattgacaaatacgatttatctaatttacacgaaattgcttctggtggcgctcc

cctctctaaggaagtcggggaagcggttgccaagaggttccatctgccaggtatcaggca

aggatatgggctcactgagactacatcagctattctgattacacccgagggggatgataa

accgggcgcggtcggtaaagttgttccattttttgaagcgaaggttgtggatctggatac

cgggaaaacgctgggcgttaatcaaagaggcgaactgtgtgtgagaggtcctatgattat

gtccggttatgtaaacaatccggaagcgaccaacgccttgattgacaaggatggatggct

acattctggagacatagcttactgggacgaagacgaacacttcttcatcgttgaccgcct

gaagtctctgattaagtacaaaggctatcaggtggctcccgctgaattggaatccatctt

gctccaacaccccaacatcttcgacgcaggtgtcgcaggtcttcccgacgatgacgccgg

tgaacttcccgccgccgttgttgttttggagcacggaaagacgatgacggaaaaagagat

cgtggattacgtcgccagtcaagtaacaaccgcgaaaaagttgcgcggaggagttgtgtt

tgtggacgaagtaccgaaaggtcttaccggaaaactcgacgcaagaaaaatcagagagat

cctcataaaggccaagaagggcggaaagatcgccgtgtaattctagatcactggatttta

ggaattagaaattttattgatagaagtattttacaaatacaaatacatactaagggtttc

ttatatgctcaacacatgagcgaaaccctataagaaccctaattcccttatctggaattc

gatatcaagcttatcgataccgtcgacctcgagggggggcccggtacccaattcgcccta

tagtgagtcgtattacaattcactggccgtcgttttacaacgtcgtgactgggaaaaccc

tggcgttacccaacttaatcgccttgcagcacatccccctttcgccagctggcgtaatag

cgaagaggcccgcaccgatcgcccttcccaacagttgcgcagcctgaatggcgaatggcg

cgaaattgtaaacgttaatgttatcgatacatgagaattaagggagtcacgttatgaccc

ccgccgatgacgcgggacaagccgttttacgtttggaactgacagaaccgcaacgttgaa

ggagccactcagccgcgggtttctggagtttaatgagctaagcacatacgtcagaaacca

ttattgcgcgttcaaaagtcgcctaaggtcactatcagctagcaaatatttcttgtcaaa

aatgctccactgacgttccataaattcccctcggtatccaattagagtctcatattcact

ctcaactcgatcgaggcatgattgaacaagatggattgcacgcaggttctccggccgctt

gggtggagaggctattcggctatgactgggcacaacagacaatcggctgctctgatgccg

ccgtgttccggctgtcagcgcaggggcgcccggttctttttgtcaagaccgacctgtccg

gtgccctgaatgaactccaagacgaggcagcgcggctatcgtggctggccacgacgggcg

ttccttgcgcagctgtgctcgacgttgtcactgaagcgggaagggactggctgctattgg

gcgaagtgccggggcaggatctcctgtcatctcaccttgctcctgccgagaaagtatcca

tcatggctgatgcaatgcggcggctgcatacgcttgatccggctacctgcccattcgacc

accaagcgaaacatcgcatcgagcgaggacgtactcggatggaagccggtcttgtcgatc

aggatgatctggacgaagagcatcaggggctcgcgccagccgaactgttcgccaggctca

aggcgcggatgcccgacggcgaggatctcgtcgtgacccagggcgatgcctgcttgccga

atatcatggtggaaaatggccgcttttctggattcatcgactgtggccggctgggtgtgg

cggaccgctatcaggacatagcgttggctacccgtgatattgctgaagagcttggcggcg

aatgggctgaccgcttcctcgtgctttacggtatcgccgctcccgattcgcagcgcatcg

ccttctatcgccttcttgacgagttcttctgagcgggactctggggttcggactctagct

agagtcaagcagatcgttcaaacatttggcaataaagtttcttaagattgaatcctgttg

ccggtcttgcgatgattatcatataatttctgttgaattacgttaagcatgtaataatta

acatgtaatgcatgacgttatttatgagatgggttttttgattagagtcccgcaattata

catttaatacgcgatagaaaacaaaatatagcgcgcaaactaggataaattatcgcgcgc

ggtgtcatctatgttactagatcgaccggcatgcaagctgataacgttacaccacaatat

atcctgccaagatctaattccggggatcggaaatccagaagcccgagaggttgccgcctt

tcgggctttttctttttcaaaaaaaaaaatttataaaacgatctgttgcggccggccgcc

gggttgtgggcaaaggcgctggcgctcgacggtgggcaaccgcttgcggttgtccacggg

cggagccggtgcgcgtagcgcattgtccacaagccaagggcgaccaataattgatatata

tattcataattgaaaagctaattgaacatactacttgctgtaactacttgccggagcgag

gggtgtttgcaagctgttgatctgaaagggctattagcgttctcacgtgcctttttgatt

agcgatttcacgtgaccttattagcgatttcacgtactccgattagcgatttcacgtacc

ctgattagcgatttcacgtggatagtttttggagcgggccggaaagccccgtgaatcaag

gctttgcggggcattagcggtttcacgtggataactaccctctatccacaggcttccggg

gataaaaa

>pCHX21-LUC

agcccgctcgacggcgggctgttggatggggatcgcctgaatcgccccatcatccagcca

gaaagtgagggagccacggttgatgagagctttgttgtaggtggaccagttggtgatttt

gaacttttgctttgccacggaacggtctgcgttgtcgggaagatgcgtgatctgatcctt

caactcagcaaaagttcgatttattcaacaaagccacgttgtgtctcaaaatctctgatg

ttacattgcacaagataaaaatatatcatcatgaacaataaaactgtctgcttacataaa

cagtaatacaaggggtgttatgagccatattcaacgggaaacgtcttgctccaggccgcg

attaaattccaacatggatgctgatttatatgggtataaatgggctcgcgataatgtcgg

gcaatcaggtgcgacaatctatggattgtatgggaagcccgatgcgccagagttgtttct

gaaacatggcaaaggtagcgttgccaatgatgttacagatgagatggtcagactaaactg

gctgacggaatttatgcctcttccgaccatcaagcattttatccgtactcctgatgatgc

atggttactcaccactgcgatcccagggaaaacagcattccaggtattagaagaatatcc

tgattcaggtgaaaatattgttgatgcgctggcagtgttcctgcgccggttgcattcgat

tcctgtttgtaattgtccttttaacagcgatcgcgtatttcgtctcgctcaggcgcaatc

acgaatgaataacggtttggttgatgcgagtgattttgatgacgagcgtaatggctggcc

tgttgaacaagtctggaaagaaatgcataaacttttgccattctcaccggattcagtcgt

cactcatggtgatttctcacttgataaccttatttttgacgaggggaaattaataggttg

tattgatgttggacgagtcggaatcgcagaccgataccaggatcttgccatcctatggaa

ctgcctcggtgagttttctccttcattacagaaacggctttttcaaaaatatggtattga

taatcctgatatgaataaattgcagtttcatttgatgctcgatgagtttttctaatcaga

attggttaattggttgtaaccatgaccaaaatcccttaacgtgagttttcgttccactga

gcgtcagaccccgtagaaaagatcaaaggatcttcttgagatcctttttttctgcgcgta

atctgctgcttgcaaacaaaaaaaccaccgctaccagcggtggtttgtttgccggatcaa

gagctaccaactctttttccgaaggtaactggcttcagcagagcgcagataccaaatact

gtccttctagtgtagccgtagttaggccaccacttcaagaactctgtagcaccgcctaca

tacctcgctctgctaatcctgttaccagtggctgctgccagtggcgataagtcgtgtctt

accgggttggactcaagacgatagttaccggataaggcgcagcggtcgggctgaacgggg

ggttcgtgcacacagcccagcttggagcgaacgacctacaccgaactgagatacctacag

cgtgagctatgagaaagcgccacgcttcccgaagggagaaaggcggacaggtatccggta

agcggcagggtcggaacaggagagcgcacgagggagcttccagggggaaacgcctggtat

ctttatagtcctgtcgggtttcgccacctctgacttgagcgtcgatttttgtgatgctcg

tcaggggggcggagcctatggaaaaacgccagcaacgcggcctttttacggttcctggcc

ttttgctggccttttgctcacatgagatctcaaacaaacacatacagcgacttagtttac

ccgccaatatatcctgtcaaggcctatcaacaagtttgtacaaaaaagcaggcttggatt

ttataatccctttataacagactaaacaagaaaaccaaccaaaattgactaatttcattt

gcaaaagagaatacaaagacagctaaattactagtacttttctatttgacatcaaacata

aaaaaaaactataattagcaatgacaaagaagaagattagagaaaaaaaaaaaaaaggaa

aaacagattgtgtagctctcacctattaatgcaataaaacaaaacagatgatgaaacaca

aagattttgtgaaagggaaaaggccaaaaccttgggttttttttgagagaatcccgttta

aatctattaaggaaaatccccccaataaatggccgatcccgcagctgaatctattgatgc

aagttcgtcgcggtttgggagagtcgtatgttataaccaaacggcaatgcatggaagcaa

caccatttcagccgcagctcctttctttatgacgcaactgtctgtcgctaatcttaccta

ccgcatactctactattttcttaagcctctatgtcttcctcctttcgtcgctcaaatact

tgtgagtagtcaatcttgaaaacatatttaactaaccattatcaatctttaaactgtttt

ttttcattacttattacccacaactattgcaaacctatgtttccttttttttctttgtta

gctatgtcctaaagagtcttcatcttcaaattcttgattaaatttttgctaacaattaac

ttcattgttctaacgaaagttcagctttttgcttcttttttttttcgaaaacggtgattt

taagataaaaccaaaatgataaaaaatttaagatttgtccacttgcaacatacgatttat

attattggatctttttcaaattaatctcgtacataatttataatatgatcctataataat

tgtaatgattattttgtttacagtgcggattactgttcagcccgactgtgctcggcaata

acgaagtggtccttaaacttatattcccttataaatatacaATGcacccagctttcttgt

acaaagtggttgccatggaagacgccaaaaacataaagaaaggcccggcgccattctatc

cgctggaagatggaaccgctggagagcaactgcataaggctatgaagagatacgccctgg

ttcctggaacaattgcttttacagatgcacatatcgaggtggacatcacttacgctgagt

acttcgaaatgtccgttcggttggcagaagctatgaaacgatatgggctgaatacaaatc

acagaatcgtcgtatgcagtgaaaactctcttcaattctttatgccggtgttgggcgcgt

tatttatcggagttgcagttgcgcccgcgaacgacatttataatgaacgtgaattgctca

acagtatgggcatttcgcagcctaccgtggtgttcgtttccaaaaaggggttgcaaaaaa

ttttgaacgtgcaaaaaaagctcccaatcatccaaaaaattattatcatggattctaaaa

cggattaccagggatttcagtcgatgtacacgttcgtcacatctcatctacctcccggtt

ttaatgaatacgattttgtgccagagtccttcgatagggacaagacaattgcactgatca

tgaactcctctggatctactggtctgcctaaaggtgtcgctctgcctcatagaactgcct

gcgtgagattctcgcatgccagagatcctatttttggcaatcaaatcattccggatactg

cgattttaagtgttgttccattccatcacggttttggaatgtttactacactcggatatt

tgatatgtggatttcgagtcgtcttaatgtatagatttgaagaagagctgtttctgagga

gccttcaggattacaagattcaaagtgcgctgctggtgccaaccctattctccttcttcg

ccaaaagcactctgattgacaaatacgatttatctaatttacacgaaattgcttctggtg

gcgctcccctctctaaggaagtcggggaagcggttgccaagaggttccatctgccaggta

tcaggcaaggatatgggctcactgagactacatcagctattctgattacacccgaggggg

atgataaaccgggcgcggtcggtaaagttgttccattttttgaagcgaaggttgtggatc

tggataccgggaaaacgctgggcgttaatcaaagaggcgaactgtgtgtgagaggtccta

tgattatgtccggttatgtaaacaatccggaagcgaccaacgccttgattgacaaggatg

gatggctacattctggagacatagcttactgggacgaagacgaacacttcttcatcgttg

accgcctgaagtctctgattaagtacaaaggctatcaggtggctcccgctgaattggaat

ccatcttgctccaacaccccaacatcttcgacgcaggtgtcgcaggtcttcccgacgatg

acgccggtgaacttcccgccgccgttgttgttttggagcacggaaagacgatgacggaaa

aagagatcgtggattacgtcgccagtcaagtaacaaccgcgaaaaagttgcgcggaggag

ttgtgtttgtggacgaagtaccgaaaggtcttaccggaaaactcgacgcaagaaaaatca

gagagatcctcataaaggccaagaagggcggaaagatcgccgtgtaattctagatcactg

gattttaggaattagaaattttattgatagaagtattttacaaatacaaatacatactaa

gggtttcttatatgctcaacacatgagcgaaaccctataagaaccctaattcccttatct

ggaattcgatatcaagcttatcgataccgtcgacctcgagggggggcccggtacccaatt

cgccctatagtgagtcgtattacaattcactggccgtcgttttacaacgtcgtgactggg

aaaaccctggcgttacccaacttaatcgccttgcagcacatccccctttcgccagctggc

gtaatagcgaagaggcccgcaccgatcgcccttcccaacagttgcgcagcctgaatggcg

aatggcgcgaaattgtaaacgttaatgttatcgatacatgagaattaagggagtcacgtt

atgacccccgccgatgacgcgggacaagccgttttacgtttggaactgacagaaccgcaa

cgttgaaggagccactcagccgcgggtttctggagtttaatgagctaagcacatacgtca

gaaaccattattgcgcgttcaaaagtcgcctaaggtcactatcagctagcaaatatttct

tgtcaaaaatgctccactgacgttccataaattcccctcggtatccaattagagtctcat

attcactctcaactcgatcgaggcatgattgaacaagatggattgcacgcaggttctccg

gccgcttgggtggagaggctattcggctatgactgggcacaacagacaatcggctgctct

gatgccgccgtgttccggctgtcagcgcaggggcgcccggttctttttgtcaagaccgac

ctgtccggtgccctgaatgaactccaagacgaggcagcgcggctatcgtggctggccacg

acgggcgttccttgcgcagctgtgctcgacgttgtcactgaagcgggaagggactggctg

ctattgggcgaagtgccggggcaggatctcctgtcatctcaccttgctcctgccgagaaa

gtatccatcatggctgatgcaatgcggcggctgcatacgcttgatccggctacctgccca

ttcgaccaccaagcgaaacatcgcatcgagcgaggacgtactcggatggaagccggtctt

gtcgatcaggatgatctggacgaagagcatcaggggctcgcgccagccgaactgttcgcc

aggctcaaggcgcggatgcccgacggcgaggatctcgtcgtgacccagggcgatgcctgc

ttgccgaatatcatggtggaaaatggccgcttttctggattcatcgactgtggccggctg

ggtgtggcggaccgctatcaggacatagcgttggctacccgtgatattgctgaagagctt

ggcggcgaatgggctgaccgcttcctcgtgctttacggtatcgccgctcccgattcgcag

cgcatcgccttctatcgccttcttgacgagttcttctgagcgggactctggggttcggac

tctagctagagtcaagcagatcgttcaaacatttggcaataaagtttcttaagattgaat

cctgttgccggtcttgcgatgattatcatataatttctgttgaattacgttaagcatgta

ataattaacatgtaatgcatgacgttatttatgagatgggttttttgattagagtcccgc

aattatacatttaatacgcgatagaaaacaaaatatagcgcgcaaactaggataaattat

cgcgcgcggtgtcatctatgttactagatcgaccggcatgcaagctgataacgttacacc

acaatatatcctgccaagatctaattccggggatcggaaatccagaagcccgagaggttg

ccgcctttcgggctttttctttttcaaaaaaaaaaatttataaaacgatctgttgcggcc

ggccgccgggttgtgggcaaaggcgctggcgctcgacggtgggcaaccgcttgcggttgt

ccacgggcggagccggtgcgcgtagcgcattgtccacaagccaagggcgaccaataattg

atatatatattcataattgaaaagctaattgaacatactacttgctgtaactacttgccg

gagcgaggggtgtttgcaagctgttgatctgaaagggctattagcgttctcacgtgcctt

tttgattagcgatttcacgtgaccttattagcgatttcacgtactccgattagcgatttc

acgtaccctgattagcgatttcacgtggatagtttttggagcgggccggaaagccccgtg

aatcaaggctttgcggggcattagcggtttcacgtggataactaccctctatccacaggc

ttccggggataaaaa

>pOPTX:LUC

agcccgctcgacggcgggctgttggatggggatcgcctgaatcgccccatcatccagcca

gaaagtgagggagccacggttgatgagagctttgttgtaggtggaccagttggtgatttt

gaacttttgctttgccacggaacggtctgcgttgtcgggaagatgcgtgatctgatcctt

caactcagcaaaagttcgatttattcaacaaagccacgttgtgtctcaaaatctctgatg

ttacattgcacaagataaaaatatatcatcatgaacaataaaactgtctgcttacataaa

cagtaatacaaggggtgttatgagccatattcaacgggaaacgtcttgctccaggccgcg

attaaattccaacatggatgctgatttatatgggtataaatgggctcgcgataatgtcgg

gcaatcaggtgcgacaatctatggattgtatgggaagcccgatgcgccagagttgtttct

gaaacatggcaaaggtagcgttgccaatgatgttacagatgagatggtcagactaaactg

gctgacggaatttatgcctcttccgaccatcaagcattttatccgtactcctgatgatgc

atggttactcaccactgcgatcccagggaaaacagcattccaggtattagaagaatatcc

tgattcaggtgaaaatattgttgatgcgctggcagtgttcctgcgccggttgcattcgat

tcctgtttgtaattgtccttttaacagcgatcgcgtatttcgtctcgctcaggcgcaatc

acgaatgaataacggtttggttgatgcgagtgattttgatgacgagcgtaatggctggcc

tgttgaacaagtctggaaagaaatgcataaacttttgccattctcaccggattcagtcgt

cactcatggtgatttctcacttgataaccttatttttgacgaggggaaattaataggttg

tattgatgttggacgagtcggaatcgcagaccgataccaggatcttgccatcctatggaa

ctgcctcggtgagttttctccttcattacagaaacggctttttcaaaaatatggtattga

taatcctgatatgaataaattgcagtttcatttgatgctcgatgagtttttctaatcaga

attggttaattggttgtaaccatgaccaaaatcccttaacgtgagttttcttccactgag

cgtcagaccccgtagaaaagatcaaaggatcttcttgagatcctttttttctgcgcgtaa

tctgctgcttgcaaacaaaaaaaccaccgctaccagcggtggtttgtttgccggatcaag

agctaccaactctttttccgaaggtaactggcttcagcagagcgcagataccaaatactg

tccttctagtgtagccgtagttaggccaccacttcaagaactctgtagcaccgcctacat

acctcgctctgctaatcctgttaccagtggctgctgccagtggcgataagtcgtgtctta

ccgggttggactcaagacgatagttaccggataaggcgcagcggtcgggctgaacggggg

gttcgtgcacacagcccagcttggagcgaacgacctacaccgaactgagatacctacagc

gtgagctatgagaaagcgccacgcttcccgaagggagaaaggcggacaggtatccggtaa

gcggcagggtcggaacaggagagcgcacgagggagcttccagggggaaacgcctggtatc

tttatagtcctgtcgggtttcgccacctctgacttgagcgtcgatttttgtgatgctcgt

caggggggcggagcctatggaaaaacgccagcaacgcggcctttttacggttcctggcct

tttgctggccttttgctcacatgagatctcaaacaaacacatacagcgacttagtttacc

cgccaatatatcctgtcaaggcctatcaacaagtttgtacaaaaaagcaggctgaaacgt

ttttcgttaaaaacttcaccgtaaattccaacaaatgtcaaacaaaaaaattatcagaac

tatccaacaaattggtcatataattattcaatggaaacgtttccaataaattcgtgataa

atgcagatttccaacgatcatattccaacaaatatatgttctattgaaaatttattggaa

acatataattcttacaaattaaccgcatagatagctgttggaattaagcttttttcttgt

agatcaaattgaaaatatttttcaccacatccatcattttcttgatttattgtatataaa

tatgtacattagatttctaattactttatcaacttttgtgaaatccataaaattttctta

aaattaaattatgatgatattgtaaacttgattagttcttttaatgttaagtttctacta

gtactgacggaaagggttgaactcacattgatgggaagggaagatcgtcaaagattgtca

tcataatcaaatcgcaactatatatattcagccgtaaatgtcaagaaaacattgtagtaa

acaatagtttatgccccagagccacaggccaaagttgaaattttcttgtaatgatgatga

tgatgatgcattttaaacatattggacagtgggtccatttcttcccactactctaataca

ctaagaagtttagtaaacttaattatttgatatatttaagttttaaattagttacacaat

ttattcaatagtattttgatatttttctaataactaaaaccataattttaaaaatgaact

tctctataagaattcattgtttctctttttacacaaaagttgatttaagaatttttttta

ctttttattagaaagatttatatttattggatattgatattgaagtctagtttctaacaa

aaattaatgtagaaatagttgattaatattggataatatttttctagtttctctttcatg

attcttttaaaaatgaacgagacattataagtacctcctcgctccccacctagttaattt

aactttgtgccaactcctaaagccaaacttatatcttcctttgtttgacataatacaaga

aaaaaagaaaagagctatggacccagctttcttgtacaaagtggttgccatggaagacgc

caaaaacataaagaaaggcccggcgccattctatccgctggaagatggaaccgctggaga

gcaactgcataaggctatgaagagatacgccctggttcctggaacaattgcttttacaga

tgcacatatcgaggtggacatcacttacgctgagtacttcgaaatgtccgttcggttggc

agaagctatgaaacgatatgggctgaatacaaatcacagaatcgtcgtatgcagtgaaaa

ctctcttcaattctttatgccggtgttgggcgcgttatttatcggagttgcagttgcgcc

cgcgaacgacatttataatgaacgtgaattgctcaacagtatgggcatttcgcagcctac

cgtggtgttcgtttccaaaaaggggttgcaaaaaattttgaacgtgcaaaaaaagctccc

aatcatccaaaaaattattatcatggattctaaaacggattaccagggatttcagtcgat

gtacacgttcgtcacatctcatctacctcccggttttaatgaatacgattttgtgccaga

gtccttcgatagggacaagacaattgcactgatcatgaactcctctggatctactggtct

gcctaaaggtgtcgctctgcctcatagaactgcctgcgtgagattctcgcatgccagaga

tcctatttttggcaatcaaatcattccggatactgcgattttaagtgttgttccattcca

tcacggttttggaatgtttactacactcggatatttgatatgtggatttcgagtcgtctt

aatgtatagatttgaagaagagctgtttctgaggagccttcaggattacaagattcaaag

tgcgctgctggtgccaaccctattctccttcttcgccaaaagcactctgattgacaaata

cgatttatctaatttacacgaaattgcttctggtggcgctcccctctctaaggaagtcgg

ggaagcggttgccaagaggttccatctgccaggtatcaggcaaggatatgggctcactga

gactacatcagctattctgattacacccgagggggatgataaaccgggcgcggtcggtaa

agttgttccattttttgaagcgaaggttgtggatctggataccgggaaaacgctgggcgt

taatcaaagaggcgaactgtgtgtgagaggtcctatgattatgtccggttatgtaaacaa

tccggaagcgaccaacgccttgattgacaaggatggatggctacattctggagacatagc

ttactgggacgaagacgaacacttcttcatcgttgaccgcctgaagtctctgattaagta

caaaggctatcaggtggctcccgctgaattggaatccatcttgctccaacaccccaacat

cttcgacgcaggtgtcgcaggtcttcccgacgatgacgccggtgaacttcccgccgccgt

tgttgttttggagcacggaaagacgatgacggaaaaagagatcgtggattacgtcgccag

tcaagtaacaaccgcgaaaaagttgcgcggaggagttgtgtttgtggacgaagtaccgaa

aggtcttaccggaaaactcgacgcaagaaaaatcagagagatcctcataaaggccaagaa

gggcggaaagatcgccgtgtaattctagatcactggattttaggaattagaaattttatt

gatagaagtattttacaaatacaaatacatactaagggtttcttatatgctcaacacatg

agcgaaaccctataagaaccctaattcccttatctggaattcgatatcaagcttatcgat

accgtcgacctcgagggggggcccggtacccaattcgccctatagtgagtcgtattacaa

ttcactggccgtcgttttacaacgtcgtgactgggaaaaccctggcgttacccaacttaa

tcgccttgcagcacatccccctttcgccagctggcgtaatagcgaagaggcccgcaccga

tcgcccttcccaacagttgcgcagcctgaatggcgaatggcgcgaaattgtaaacgttaa

tgttatcgatacatgagaattaagggagtcacgttatgacccccgccgatgacgcgggac

aagccgttttacgtttggaactgacagaaccgcaacgttgaaggagccactcagccgcgg

gtttctggagtttaatgagctaagcacatacgtcagaaaccattattgcgcgttcaaaag

tcgcctaaggtcactatcagctagcaaatatttcttgtcaaaaatgctccactgacgttc

cataaattcccctcggtatccaattagagtctcatattcactctcaactcgatcgaggca

tgattgaacaagatggattgcacgcaggttctccggccgcttgggtggagaggctattcg

gctatgactgggcacaacagacaatcggctgctctgatgccgccgtgttccggctgtcag

cgcaggggcgcccggttctttttgtcaagaccgacctgtccggtgccctgaatgaactcc

aagacgaggcagcgcggctatcgtggctggccacgacgggcgttccttgcgcagctgtgc

tcgacgttgtcactgaagcgggaagggactggctgctattgggcgaagtgccggggcagg

atctcctgtcatctcaccttgctcctgccgagaaagtatccatcatggctgatgcaatgc

ggcggctgcatacgcttgatccggctacctgcccattcgaccaccaagcgaaacatcgca

tcgagcgaggacgtactcggatggaagccggtcttgtcgatcaggatgatctggacgaag

agcatcaggggctcgcgccagccgaactgttcgccaggctcaaggcgcggatgcccgacg

gcgaggatctcgtcgtgacccagggcgatgcctgcttgccgaatatcatggtggaaaatg

gccgcttttctggattcatcgactgtggccggctgggtgtggcggaccgctatcaggaca

tagcgttggctacccgtgatattgctgaagagcttggcggcgaatgggctgaccgcttcc

tcgtgctttacggtatcgccgctcccgattcgcagcgcatcgccttctatcgccttcttg

acgagttcttctgagcgggactctggggttcggactctagctagagtcaagcagatcgtt

caaacatttggcaataaagtttcttaagattgaatcctgttgccggtcttgcgatgatta

tcatataatttctgttgaattacgttaagcatgtaataattaacatgtaatgcatgacgt

tatttatgagatgggttttttgattagagtcccgcaattatacatttaatacgcgataga

aaacaaaatatagcgcgcaaactaggataaattatcgcgcgcggtgtcatctatgttact

agatcgaccggcatgcaagctgataacgttacaccacaatatatcctgccaagatctaat

tccggggatcggaaatccagaagcccgagaggttgccgcctttcgggctttttctttttc

aaaaaaaaaaatttataaaacgatctgttgcggccggccgccgggttgtgggcaaaggcg

ctggcgctcgacggtgggcaaccgcttgcggttgtccacgggcggagccggtgcgcgtag

cgcattgtccacaagccaagggcgaccaataattgatatatatattcataattgaaaagc

taattgaacatactacttgctgtaactacttgccggagcgaggggtgtttgcaagctgtt

gatctgaaagggctattagcgttctcacgtgcctttttgattagcgatttcacgtgacct

tattagcgatttcacgtactccgattagcgatttcacgtaccctgattagcgatttcacg

tggatagtttttggagcgggccggaaagccccgtgaatcaaggctttgcggggcattagc

ggtttcacgtggataactaccctctatccacaggcttccggggataaaaa

>pOPTXCGMP:LUC

agcccgctcgacggcgggctgttggatggggatcgcctgaatcgccccatcatccagcca

gaaagtgagggagccacggttgatgagagctttgttgtaggtggaccagttggtgatttt

gaacttttgctttgccacggaacggtctgcgttgtcgggaagatgcgtgatctgatcctt

caactcagcaaaagttcgatttattcaacaaagccacgttgtgtctcaaaatctctgatg

ttacattgcacaagataaaaatatatcatcatgaacaataaaactgtctgcttacataaa

cagtaatacaaggggtgttatgagccatattcaacgggaaacgtcttgctccaggccgcg

attaaattccaacatggatgctgatttatatgggtataaatgggctcgcgataatgtcgg

gcaatcaggtgcgacaatctatggattgtatgggaagcccgatgcgccagagttgtttct

gaaacatggcaaaggtagcgttgccaatgatgttacagatgagatggtcagactaaactg

gctgacggaatttatgcctcttccgaccatcaagcattttatccgtactcctgatgatgc

atggttactcaccactgcgatcccagggaaaacagcattccaggtattagaagaatatcc

tgattcaggtgaaaatattgttgatgcgctggcagtgttcctgcgccggttgcattcgat

tcctgtttgtaattgtccttttaacagcgatcgcgtatttcgtctcgctcaggcgcaatc

acgaatgaataacggtttggttgatgcgagtgattttgatgacgagcgtaatggctggcc

tgttgaacaagtctggaaagaaatgcataaacttttgccattctcaccggattcagtcgt

cactcatggtgatttctcacttgataaccttatttttgacgaggggaaattaataggttg

tattgatgttggacgagtcggaatcgcagaccgataccaggatcttgccatcctatggaa

ctgcctcggtgagttttctccttcattacagaaacggctttttcaaaaatatggtattga

taatcctgatatgaataaattgcagtttcatttgatgctcgatgagtttttctaatcaga

attggttaattggttgtaaccatgaccaaaatcccttaacgtgagttttcttccactgag

cgtcagaccccgtagaaaagatcaaaggatcttcttgagatcctttttttctgcgcgtaa

tctgctgcttgcaaacaaaaaaaccaccgctaccagcggtggtttgtttgccggatcaag

agctaccaactctttttccgaaggtaactggcttcagcagagcgcagataccaaatactg

tccttctagtgtagccgtagttaggccaccacttcaagaactctgtagcaccgcctacat

acctcgctctgctaatcctgttaccagtggctgctgccagtggcgataagtcgtgtctta

ccgggttggactcaagacgatagttaccggataaggcgcagcggtcgggctgaacggggg

gttcgtgcacacagcccagcttggagcgaacgacctacaccgaactgagatacctacagc

gtgagctatgagaaagcgccacgcttcccgaagggagaaaggcggacaggtatccggtaa

gcggcagggtcggaacaggagagcgcacgagggagcttccagggggaaacgcctggtatc

tttatagtcctgtcgggtttcgccacctctgacttgagcgtcgatttttgtgatgctcgt

caggggggcggagcctatggaaaaacgccagcaacgcggcctttttacggttcctggcct

tttgctggccttttgctcacatgagatctcaaacaaacacatacagcgacttagtttacc

cgccaatatatcctgtcaaggcctatcaacaagtttgtacaaaaaagcaggctgaaacgt

ttttcgttaaaaacttcaccgtaaattccaacaaatgtcaaacaaaaaaattatcagaac

tatccaacaaattggtcatataattattcaatggaaacgtttccaataaattcgtgataa

atgcagatttccaacgatcatattccaacaaatatatgttctattgaaaatttattggaa

acatataattcttacaaattaaccgcatagatagctgttggaattaagcttttttcttgt

agatcaaattgaaaatatttttcaccacatccatcattttcttgatttattgtatataaa

tatgtacattagatttctaattactttatcaacttttgtgaaatccataaaattttctta

aaattaaattatgatgatattgtaaacttgattagttcttttaatgttaagtttctacta

gtactgacggaaagggttgaactcacattgatgggaagggaagatcgtcaaagattgtca

tcataatcaaatcgcaactatatatattcagccgtaaatgtcaagaaaacattgtagtaa

acaatagtttatgccccagagccacaggccaaagttgaaattttcttgtaatgatgatga

tgatgatgcattttaaacatattggacagtgggtccatttcttcccactactctaataca

ctaagaagtttagtaaacttaattatttgatatatttaagttttaaattagttacacaat

ttattcaatagtattttgatatttttctaataactaaaaccataattttaaaaatgaact

tctctataagaattcattgtttctctttttacacaaaagttgatttaagaatttttttta

ctttttattagaaagatttatatttattggatattgatattgaagtctagtttctaacaa

aaattccAaatagatttcaacagttgagagccAaatagatttcaacagttgagagccAaa

tagatttcaacagttaatgtagaaatagttgattaatattggataatatttttctagttt

ctctttcatgattcttttaaaaatgaacgagacattataagtacctcctcgctccccacc

tagttaatttaactttgtgccaactcctaaagccaaacttatatcttcctttgtttgaca

taatacaagaaaaaaagaaaagagctATGgacccagctttcttgtacaaagtggttgcca

tggaagacgccaaaaacataaagaaaggcccggcgccattctatccgctggaagatggaa

ccgctggagagcaactgcataaggctatgaagagatacgccctggttcctggaacaattg

cttttacagatgcacatatcgaggtggacatcacttacgctgagtacttcgaaatgtccg

ttcggttggcagaagctatgaaacgatatgggctgaatacaaatcacagaatcgtcgtat

gcagtgaaaactctcttcaattctttatgccggtgttgggcgcgttatttatcggagttg

cagttgcgcccgcgaacgacatttataatgaacgtgaattgctcaacagtatgggcattt

cgcagcctaccgtggtgttcgtttccaaaaaggggttgcaaaaaattttgaacgtgcaaa

aaaagctcccaatcatccaaaaaattattatcatggattctaaaacggattaccagggat

ttcagtcgatgtacacgttcgtcacatctcatctacctcccggttttaatgaatacgatt

ttgtgccagagtccttcgatagggacaagacaattgcactgatcatgaactcctctggat

ctactggtctgcctaaaggtgtcgctctgcctcatagaactgcctgcgtgagattctcgc

atgccagagatcctatttttggcaatcaaatcattccggatactgcgattttaagtgttg

ttccattccatcacggttttggaatgtttactacactcggatatttgatatgtggatttc

gagtcgtcttaatgtatagatttgaagaagagctgtttctgaggagccttcaggattaca

agattcaaagtgcgctgctggtgccaaccctattctccttcttcgccaaaagcactctga

ttgacaaatacgatttatctaatttacacgaaattgcttctggtggcgctcccctctcta

aggaagtcggggaagcggttgccaagaggttccatctgccaggtatcaggcaaggatatg

ggctcactgagactacatcagctattctgattacacccgagggggatgataaaccgggcg

cggtcggtaaagttgttccattttttgaagcgaaggttgtggatctggataccgggaaaa

cgctgggcgttaatcaaagaggcgaactgtgtgtgagaggtcctatgattatgtccggtt

atgtaaacaatccggaagcgaccaacgccttgattgacaaggatggatggctacattctg

gagacatagcttactgggacgaagacgaacacttcttcatcgttgaccgcctgaagtctc

tgattaagtacaaaggctatcaggtggctcccgctgaattggaatccatcttgctccaac

accccaacatcttcgacgcaggtgtcgcaggtcttcccgacgatgacgccggtgaacttc

ccgccgccgttgttgttttggagcacggaaagacgatgacggaaaaagagatcgtggatt

acgtcgccagtcaagtaacaaccgcgaaaaagttgcgcggaggagttgtgtttgtggacg

aagtaccgaaaggtcttaccggaaaactcgacgcaagaaaaatcagagagatcctcataa

aggccaagaagggcggaaagatcgccgtgtaattctagatcactggattttaggaattag

aaattttattgatagaagtattttacaaatacaaatacatactaagggtttcttatatgc

tcaacacatgagcgaaaccctataagaaccctaattcccttatctggaattcgatatcaa

gcttatcgataccgtcgacctcgagggggggcccggtacccaattcgccctatagtgagt

cgtattacaattcactggccgtcgttttacaacgtcgtgactgggaaaaccctggcgtta

cccaacttaatcgccttgcagcacatccccctttcgccagctggcgtaatagcgaagagg

cccgcaccgatcgcccttcccaacagttgcgcagcctgaatggcgaatggcgcgaaattg

taaacgttaatgttatcgatacatgagaattaagggagtcacgttatgacccccgccgat

gacgcgggacaagccgttttacgtttggaactgacagaaccgcaacgttgaaggagccac

tcagccgcgggtttctggagtttaatgagctaagcacatacgtcagaaaccattattgcg

cgttcaaaagtcgcctaaggtcactatcagctagcaaatatttcttgtcaaaaatgctcc

actgacgttccataaattcccctcggtatccaattagagtctcatattcactctcaactc

gatcgaggcatgattgaacaagatggattgcacgcaggttctccggccgcttgggtggag

aggctattcggctatgactgggcacaacagacaatcggctgctctgatgccgccgtgttc

cggctgtcagcgcaggggcgcccggttctttttgtcaagaccgacctgtccggtgccctg

aatgaactccaagacgaggcagcgcggctatcgtggctggccacgacgggcgttccttgc

gcagctgtgctcgacgttgtcactgaagcgggaagggactggctgctattgggcgaagtg

ccggggcaggatctcctgtcatctcaccttgctcctgccgagaaagtatccatcatggct

gatgcaatgcggcggctgcatacgcttgatccggctacctgcccattcgaccaccaagcg

aaacatcgcatcgagcgaggacgtactcggatggaagccggtcttgtcgatcaggatgat

ctggacgaagagcatcaggggctcgcgccagccgaactgttcgccaggctcaaggcgcgg

atgcccgacggcgaggatctcgtcgtgacccagggcgatgcctgcttgccgaatatcatg

gtggaaaatggccgcttttctggattcatcgactgtggccggctgggtgtggcggaccgc

tatcaggacatagcgttggctacccgtgatattgctgaagagcttggcggcgaatgggct

gaccgcttcctcgtgctttacggtatcgccgctcccgattcgcagcgcatcgccttctat

cgccttcttgacgagttcttctgagcgggactctggggttcggactctagctagagtcaa

gcagatcgttcaaacatttggcaataaagtttcttaagattgaatcctgttgccggtctt

gcgatgattatcatataatttctgttgaattacgttaagcatgtaataattaacatgtaa

tgcatgacgttatttatgagatgggttttttgattagagtcccgcaattatacatttaat

acgcgatagaaaacaaaatatagcgcgcaaactaggataaattatcgcgcgcggtgtcat

ctatgttactagatcgaccggcatgcaagctgataacgttacaccacaatatatcctgcc

aagatctaattccggggatcggaaatccagaagcccgagaggttgccgcctttcgggctt

tttctttttcaaaaaaaaaaatttataaaacgatctgttgcggccggccgccgggttgtg

ggcaaaggcgctggcgctcgacggtgggcaaccgcttgcggttgtccacgggcggagccg

gtgcgcgtagcgcattgtccacaagccaagggcgaccaataattgatatatatattcata

attgaaaagctaattgaacatactacttgctgtaactacttgccggagcgaggggtgttt

gcaagctgttgatctgaaagggctattagcgttctcacgtgcctttttgattagcgattt

cacgtgaccttattagcgatttcacgtactccgattagcgatttcacgtaccctgattag

cgatttcacgtggatagtttttggagcgggccggaaagccccgtgaatcaaggctttgcg

gggcattagcggtttcacgtggataactaccctctatccacaggcttccggggataaaaa

>OPTXGARE:LUC

agcccgctcgacggcgggctgttggatggggatcgcctgaatcgccccatcatccagcca

gaaagtgagggagccacggttgatgagagctttgttgtaggtggaccagttggtgatttt

gaacttttgctttgccacggaacggtctgcgttgtcgggaagatgcgtgatctgatcctt

caactcagcaaaagttcgatttattcaacaaagccacgttgtgtctcaaaatctctgatg

ttacattgcacaagataaaaatatatcatcatgaacaataaaactgtctgcttacataaa

cagtaatacaaggggtgttatgagccatattcaacgggaaacgtcttgctccaggccgcg

attaaattccaacatggatgctgatttatatgggtataaatgggctcgcgataatgtcgg

gcaatcaggtgcgacaatctatggattgtatgggaagcccgatgcgccagagttgtttct

gaaacatggcaaaggtagcgttgccaatgatgttacagatgagatggtcagactaaactg

gctgacggaatttatgcctcttccgaccatcaagcattttatccgtactcctgatgatgc

atggttactcaccactgcgatcccagggaaaacagcattccaggtattagaagaatatcc

tgattcaggtgaaaatattgttgatgcgctggcagtgttcctgcgccggttgcattcgat

tcctgtttgtaattgtccttttaacagcgatcgcgtatttcgtctcgctcaggcgcaatc

acgaatgaataacggtttggttgatgcgagtgattttgatgacgagcgtaatggctggcc

tgttgaacaagtctggaaagaaatgcataaacttttgccattctcaccggattcagtcgt

cactcatggtgatttctcacttgataaccttatttttgacgaggggaaattaataggttg

tattgatgttggacgagtcggaatcgcagaccgataccaggatcttgccatcctatggaa

ctgcctcggtgagttttctccttcattacagaaacggctttttcaaaaatatggtattga

taatcctgatatgaataaattgcagtttcatttgatgctcgatgagtttttctaatcaga

attggttaattggttgtaaccatgaccaaaatcccttaacgtgagttttcttccactgag

cgtcagaccccgtagaaaagatcaaaggatcttcttgagatcctttttttctgcgcgtaa

tctgctgcttgcaaacaaaaaaaccaccgctaccagcggtggtttgtttgccggatcaag

agctaccaactctttttccgaaggtaactggcttcagcagagcgcagataccaaatactg

tccttctagtgtagccgtagttaggccaccacttcaagaactctgtagcaccgcctacat

acctcgctctgctaatcctgttaccagtggctgctgccagtggcgataagtcgtgtctta

ccgggttggactcaagacgatagttaccggataaggcgcagcggtcgggctgaacggggg

gttcgtgcacacagcccagcttggagcgaacgacctacaccgaactgagatacctacagc

gtgagctatgagaaagcgccacgcttcccgaagggagaaaggcggacaggtatccggtaa

gcggcagggtcggaacaggagagcgcacgagggagcttccagggggaaacgcctggtatc

tttatagtcctgtcgggtttcgccacctctgacttgagcgtcgatttttgtgatgctcgt

caggggggcggagcctatggaaaaacgccagcaacgcggcctttttacggttcctggcct

tttgctggccttttgctcacatgagatctcaaacaaacacatacagcgacttagtttacc

cgccaatatatcctgtcaaggcctatcaacaagtttgtacaaaaaagcaggctgaaacgt

ttttcgttaaaaacttcaccgtaaattccaacaaatgtcaaacaaaaaaattatcagaac

tatccaacaaattggtcatataattattcaatggaaacgtttccaataaattcgtgataa

atgcagatttccaacgatcatattccaacaaatatatgttctattgaaaatttattggaa

acatataattcttacaaattaaccgcatagatagctgttggaattaagcttttttcttgt

agatcaaattgaaaatatttttcaccacatccatcattttcttgatttattgtatataaa

tatgtacattagatttctaattactttatcaacttttgtgaaatccataaaattttctta

aaattaaattatgatgatattgtaaacttgattagttcttttaatgttaagtttctacta

gtactgacggaaagggttgaactcacattgatgggaagggaagatcgtcaaagattgtca

tcataatcaaatcgcaactatatatattcagccgtaaatgtcaagaaaacattgtagtaa

acaatagtttatgccccagagccacaggccaaagttgaaattttcttgtaatgatgatga

tgatgatgcattttaaacatattggacagtgggtccatttcttcccactactctaataca

ctaagaagtttagtaaacttaattatttgatatatttaagttttaaattagttacacaat

ttattcaatagtattttgatatttttctaataactaaaaccataattttaaaaatgaact

tctctataagaattcattgtttctctttttacacaaaagttgatttaagaatttttttta

ctttttattagaaagatttatatttattggatattgatattgaagtctagtttctaacaa

aaattcctaacaaagagAgcctaacaaagagAgcctaacaaagagAgcctaacaaagagA

gcctaacaaagagaatgtagaaatagttgattaatattggataatatttttctagtttct

ctttcatgattcttttaaaaatgaacgagacattataagtacctcctcgctccccaccta

gttaatttaactttgtgccaactcctaaagccaaacttatatcttcctttgtttgacata

atacaagaaaaaaagaaaagagctATGgacccagctttcttgtacaaagtggttgccatg

gaagacgccaaaaacataaagaaaggcccggcgccattctatccgctggaagatggaacc

gctggagagcaactgcataaggctatgaagagatacgccctggttcctggaacaattgct

tttacagatgcacatatcgaggtggacatcacttacgctgagtacttcgaaatgtccgtt

cggttggcagaagctatgaaacgatatgggctgaatacaaatcacagaatcgtcgtatgc

agtgaaaactctcttcaattctttatgccggtgttgggcgcgttatttatcggagttgca

gttgcgcccgcgaacgacatttataatgaacgtgaattgctcaacagtatgggcatttcg

cagcctaccgtggtgttcgtttccaaaaaggggttgcaaaaaattttgaacgtgcaaaaa

aagctcccaatcatccaaaaaattattatcatggattctaaaacggattaccagggattt

cagtcgatgtacacgttcgtcacatctcatctacctcccggttttaatgaatacgatttt

gtgccagagtccttcgatagggacaagacaattgcactgatcatgaactcctctggatct

actggtctgcctaaaggtgtcgctctgcctcatagaactgcctgcgtgagattctcgcat

gccagagatcctatttttggcaatcaaatcattccggatactgcgattttaagtgttgtt

ccattccatcacggttttggaatgtttactacactcggatatttgatatgtggatttcga

gtcgtcttaatgtatagatttgaagaagagctgtttctgaggagccttcaggattacaag

attcaaagtgcgctgctggtgccaaccctattctccttcttcgccaaaagcactctgatt

gacaaatacgatttatctaatttacacgaaattgcttctggtggcgctcccctctctaag

gaagtcggggaagcggttgccaagaggttccatctgccaggtatcaggcaaggatatggg

ctcactgagactacatcagctattctgattacacccgagggggatgataaaccgggcgcg

gtcggtaaagttgttccattttttgaagcgaaggttgtggatctggataccgggaaaacg

ctgggcgttaatcaaagaggcgaactgtgtgtgagaggtcctatgattatgtccggttat

gtaaacaatccggaagcgaccaacgccttgattgacaaggatggatggctacattctgga

gacatagcttactgggacgaagacgaacacttcttcatcgttgaccgcctgaagtctctg

attaagtacaaaggctatcaggtggctcccgctgaattggaatccatcttgctccaacac

cccaacatcttcgacgcaggtgtcgcaggtcttcccgacgatgacgccggtgaacttccc

gccgccgttgttgttttggagcacggaaagacgatgacggaaaaagagatcgtggattac

gtcgccagtcaagtaacaaccgcgaaaaagttgcgcggaggagttgtgtttgtggacgaa

gtaccgaaaggtcttaccggaaaactcgacgcaagaaaaatcagagagatcctcataaag

gccaagaagggcggaaagatcgccgtgtaattctagatcactggattttaggaattagaa

attttattgatagaagtattttacaaatacaaatacatactaagggtttcttatatgctc

aacacatgagcgaaaccctataagaaccctaattcccttatctggaattcgatatcaagc

ttatcgataccgtcgacctcgagggggggcccggtacccaattcgccctatagtgagtcg

tattacaattcactggccgtcgttttacaacgtcgtgactgggaaaaccctggcgttacc

caacttaatcgccttgcagcacatccccctttcgccagctggcgtaatagcgaagaggcc

cgcaccgatcgcccttcccaacagttgcgcagcctgaatggcgaatggcgcgaaattgta

aacgttaatgttatcgatacatgagaattaagggagtcacgttatgacccccgccgatga

cgcgggacaagccgttttacgtttggaactgacagaaccgcaacgttgaaggagccactc

agccgcgggtttctggagtttaatgagctaagcacatacgtcagaaaccattattgcgcg

ttcaaaagtcgcctaaggtcactatcagctagcaaatatttcttgtcaaaaatgctccac

tgacgttccataaattcccctcggtatccaattagagtctcatattcactctcaactcga

tcgaggcatgattgaacaagatggattgcacgcaggttctccggccgcttgggtggagag

gctattcggctatgactgggcacaacagacaatcggctgctctgatgccgccgtgttccg

gctgtcagcgcaggggcgcccggttctttttgtcaagaccgacctgtccggtgccctgaa

tgaactccaagacgaggcagcgcggctatcgtggctggccacgacgggcgttccttgcgc

agctgtgctcgacgttgtcactgaagcgggaagggactggctgctattgggcgaagtgcc

ggggcaggatctcctgtcatctcaccttgctcctgccgagaaagtatccatcatggctga

tgcaatgcggcggctgcatacgcttgatccggctacctgcccattcgaccaccaagcgaa

acatcgcatcgagcgaggacgtactcggatggaagccggtcttgtcgatcaggatgatct

ggacgaagagcatcaggggctcgcgccagccgaactgttcgccaggctcaaggcgcggat

gcccgacggcgaggatctcgtcgtgacccagggcgatgcctgcttgccgaatatcatggt

ggaaaatggccgcttttctggattcatcgactgtggccggctgggtgtggcggaccgcta

tcaggacatagcgttggctacccgtgatattgctgaagagcttggcggcgaatgggctga

ccgcttcctcgtgctttacggtatcgccgctcccgattcgcagcgcatcgccttctatcg

ccttcttgacgagttcttctgagcgggactctggggttcggactctagctagagtcaagc

agatcgttcaaacatttggcaataaagtttcttaagattgaatcctgttgccggtcttgc

gatgattatcatataatttctgttgaattacgttaagcatgtaataattaacatgtaatg

catgacgttatttatgagatgggttttttgattagagtcccgcaattatacatttaatac

gcgatagaaaacaaaatatagcgcgcaaactaggataaattatcgcgcgcggtgtcatct

atgttactagatcgaccggcatgcaagctgataacgttacaccacaatatatcctgccaa

gatctaattccggggatcggaaatccagaagcccgagaggttgccgcctttcgggctttt

tctttttcaaaaaaaaaaatttataaaacgatctgttgcggccggccgccgggttgtggg

caaaggcgctggcgctcgacggtgggcaaccgcttgcggttgtccacgggcggagccggt

gcgcgtagcgcattgtccacaagccaagggcgaccaataattgatatatatattcataat

tgaaaagctaattgaacatactacttgctgtaactacttgccggagcgaggggtgtttgc

aagctgttgatctgaaagggctattagcgttctcacgtgcctttttgattagcgatttca

cgtgaccttattagcgatttcacgtactccgattagcgatttcacgtaccctgattagcg

atttcacgtggatagtttttggagcgggccggaaagccccgtgaatcaaggctttgcggg

gcattagcggtttcacgtggataactaccctctatccacaggcttccggggataaaaa
