# Supplementary material for: A cyclic nucleotide sensitive promoter reporter system suitable for bacteria and plant cells
Source: BMC Biotechnol. 2013 Nov 9;13:97. doi: 10.1186/1472-6750-13-97 (PMC3829209; doi:10.1186/1472-6750-13-97)
Supplement: Additional file 2: Table S1 — Primer sequences used in this study. [file 1472-6750-13-97-S2.docx]

**Additional file 2: Table S1.** Primer sequences used in this study

| **Primer name** | **Primer sequence (5’ to 3’)** |
| --- | --- |
| OPTX-for-RT | ctgattcataccttggaagc |
| OPTX-rev-RT | tcctcaaatctttcctttga |
| CHX21-for-RT | tccacctagtcgagctaacgg |
| CHX21-rev-RT | tgttgttttctgcggtttgtc |
| SOS3-for-RT | aagaagaagaagaagaatgca |
| SOS3-rev-RT | gaaacgaaacttggaaacgtc c |
| OPTX -P-fwd | cactttgcattccccacac |
| OPTX -P-rev | tgattcctccgacgtgtgta |
| CHX21-P-fwd | gccctttacgtttgtttcca |
| CHX21-P-rev | cttgagatccgcaagaatcc |
| SOS3-P-fwd | gctgttagctggagcggtaa |
| SOS3-P-REV | gccagctgaaattcttcctg |
| OPTX -P-F-GW | ggggacaagtttgtacaaaaaagcaggctgaaacgtttttcgttaaaaacttc |
| OPTX -P-R-GW | ggggaccactttgtacaagaaagctgggtccatagctcttttctttttttcttgt |
| CHX21-P-F-GW | ggggacaagtttgtacaaaaaagcaggcttggattttataatccctttataac |
| CHX21-P-R-GW | ggggaccactttgtacaagaaagctgggtgcattgtatatttataagggaatataag |
| SOS3-P-F-GW | ggggacaagtttgtacaaaaaagcaggctagatttgatattgatccggtatt |
| SOS3-P-R-GW | ggggaccactttgtacaagaaagctgggtccatacaaacacacccttctctcaactg |
| OPTX -GARE-fwd | [phos]cctaacaaagagagcctaacaaagagagcctaacaaagagagcctaacaaagagagcctaacaaagagaatgtagaaatagttgattaatattggataatatttt |
| OPTX -cGMP RE-fwd | [phos]ccaaatagatttcaacagttgagagccaaatagatttcaacagttgagccaaatagatttcaacagttaatgtagaaatagttgattaatattggataatatttttctagtttctct |
| OPTX -RE-rev | [phos]aatttttgttagaaactagacttcaatatcaatatcc |
